# Multiple Measures of Adiposity Are Associated with Mean Leukocyte Telomere Length in the Northern Finland Birth Cohort 1966

**DOI:** 10.1371/journal.pone.0099133

**Published:** 2014-06-11

**Authors:** Jessica L. Buxton, Shikta Das, Alina Rodriguez, Marika Kaakinen, Alexessander Couto Alves, Sylvain Sebert, Iona Y. Millwood, Jaana Laitinen, Paul F. O’Reilly, Marjo-Riitta Jarvelin, Alexandra I. F. Blakemore

**Affiliations:** 1 Section of Investigative Medicine, Department of Medicine, Imperial College London, London, United Kingdom; 2 Department of Epidemiology and Biostatistics, Medical Research Council (MRC) Public Health England (PHE) Centre for Environment and Health, School of Public Health, Imperial College London, London, United Kingdom; 3 Department of Primary Care and Public Health, School of Public Health, Imperial College London, London, United Kingdom; 4 Department of Psychology, Mid Sweden University, Östersund, Sweden; 5 Institute of Health Sciences, University of Oulu, Oulu, Finland; 6 Biocenter Oulu, University of Oulu, Oulu, Finland; 7 Clinical Trial Service Unit and Epidemiological Studies Unit (CTSU), University of Oxford, Oxford, United Kingdom; 8 Finnish Institute of Occupational Health, Helsinki, Finland; 9 MRC Social, Genetic and Developmental Psychiatry Centre, Institute of Psychiatry, King’s College London, London, United Kingdom; 10 Department of Children and Young People and Families, National Institute for Health and Welfare, Oulu, Finland; 11 Unit of Primary Care, University of Oulu, Oulu, Finland; Newcastle University, United Kingdom

## Abstract

Studies of leukocyte telomere length (LTL) and adiposity have produced conflicting results, and the relationship between body mass index (BMI) and telomere length throughout life remains unclear. We therefore tested association of adult LTL measured in 5,598 participants with: i) childhood growth measures (BMI and age at adiposity rebound (AR)); ii) change in BMI from childhood to adulthood and iii) adult BMI, waist-to-hip ratio (WHR), body adiposity index (BAI). Childhood BMI at AR was positively associated with LTL at 31 years in women (*P* = 0.041). Adult BMI and WHR in both men (*P* = 0.025 and *P* = 0.049, respectively) and women (*P* = 0.029 and *P* = 0.008, respectively), and BAI in women (*P* = 0.021) were inversely associated with LTL at 31 years. An increase in standardised BMI between early childhood and adulthood was associated with shorter adult LTL in women (*P* = 0.008). We show that LTL is inversely associated with multiple measures of adiposity in both men and women. Additionally, BMI increase in women from childhood to adulthood is associated with shorter telomeres at age 31, potentially indicating accelerated biological ageing.

## Introduction

Telomeres are protective DNA-protein structures that cap the ends of linear chromosomes, extending to 10–15 kb in humans. In proliferative tissues such as leukocytes, telomeres shorten with each cell division – a process believed to be accelerated by oxidative stress and inflammation [1]–[3]. Mean leukocyte telomere length (LTL) in adulthood is influenced by genetic factors [4], and multiple environmental exposures including chronic psychological stress [5], childhood adversities [6], [7] and lifestyle factors such as smoking [8]. LTL is positively correlated with human lifespan [9], while shorter LTL is associated with several age-related conditions including type 2 diabetes (T2D), heart disease and some cancers [10]–[13]. LTL has thus been proposed as a biomarker of biological ageing, though further longitudinal studies are required to confirm this hypothesis [14], [15].

Inverse associations between telomere length and body mass index (BMI), waist-to-hip ratio (WHR), waist circumference and visceral fat in adulthood have been reported [16]–[20]. However, other studies have indicated a lack of association with these adiposity-related phenotypes (reviewed in [21]). Furthermore, the relationship between telomere length and body adiposity index (BAI, calculated as (hip circumference (cm))/((height(m))^1.5^)−18) has not been investigated, despite assertions that this anthropometric measure may be a better index of body fat than BMI [22]. One explanation for the conflicting results for associations between LTL and measures of adiposity could be sample diversity both within and between studies, in particular the inclusion of wide age-ranges and uneven sex distributions. Indeed, one study reports a stronger inverse association between BMI and LTL in young (<30 years) compared to older (>60 years) women [16], suggesting that the relationship between obesity and telomere length may change throughout life. In recognition of these inconsistencies in the current literature, a recent review and meta-analysis of previous studies of telomere length and BMI called for further large-scale epidemiological studies in different age groups and sexes, ideally including additional measures of adiposity [23]. Our objective was to address this gap in research.

With regard to childhood obesity, we previously carried out a case-control study in a cohort aged 2–17 years. This analysis revealed that severely obese children have shorter white blood cell telomeres in childhood than their normal weight peers [24]. However, to our knowledge, no workers have investigated whether childhood BMI, or the increase in BMI from childhood to adulthood, is associated with telomere length in adulthood. Given the rising prevalence of both child and adult obesity worldwide, and the potential increased risk of age-related conditions such as T2D and cardiovascular disease that is associated with excess or abnormal fat accumulation, these are key issues to address [25], [26]. The biological mechanisms that may underlie associations between shorter LTL and increased BMI are currently unknown, although obesity is recognised as a state of both increased oxidative stress and low-grade inflammation: both processes that are believed to accelerate leukocyte telomere attrition [1]–[3].

In the present study, we aimed to investigate the relationship between telomere length and adiposity using a longitudinal design, making use of prospectively gathered data from the Northern Finland Birth Cohort 1966 (NFBC 1966). We used childhood growth data to model age and BMI at adiposity rebound (AR), i.e. the point at which BMI reaches its nadir in early childhood (∼5 years). Early AR has been shown to predict increased adiposity in adolescence [27] and may also be related to poorer metabolic health in adulthood [28]. Using anthropometric and telomere measurement data obtained for 5,598 participants aged 31 (48% male), our aims were threefold:

- To investigate the association between age/BMI at AR in childhood and LTL at age 31;
- To investigate the association between longitudinal change in BMI from childhood (∼5 years) to age 31 and LTL;
- To test the association between WHR, BMI, BAI and LTL at age 31.

## Materials and Methods

### Study Samples

**Figure 1:**
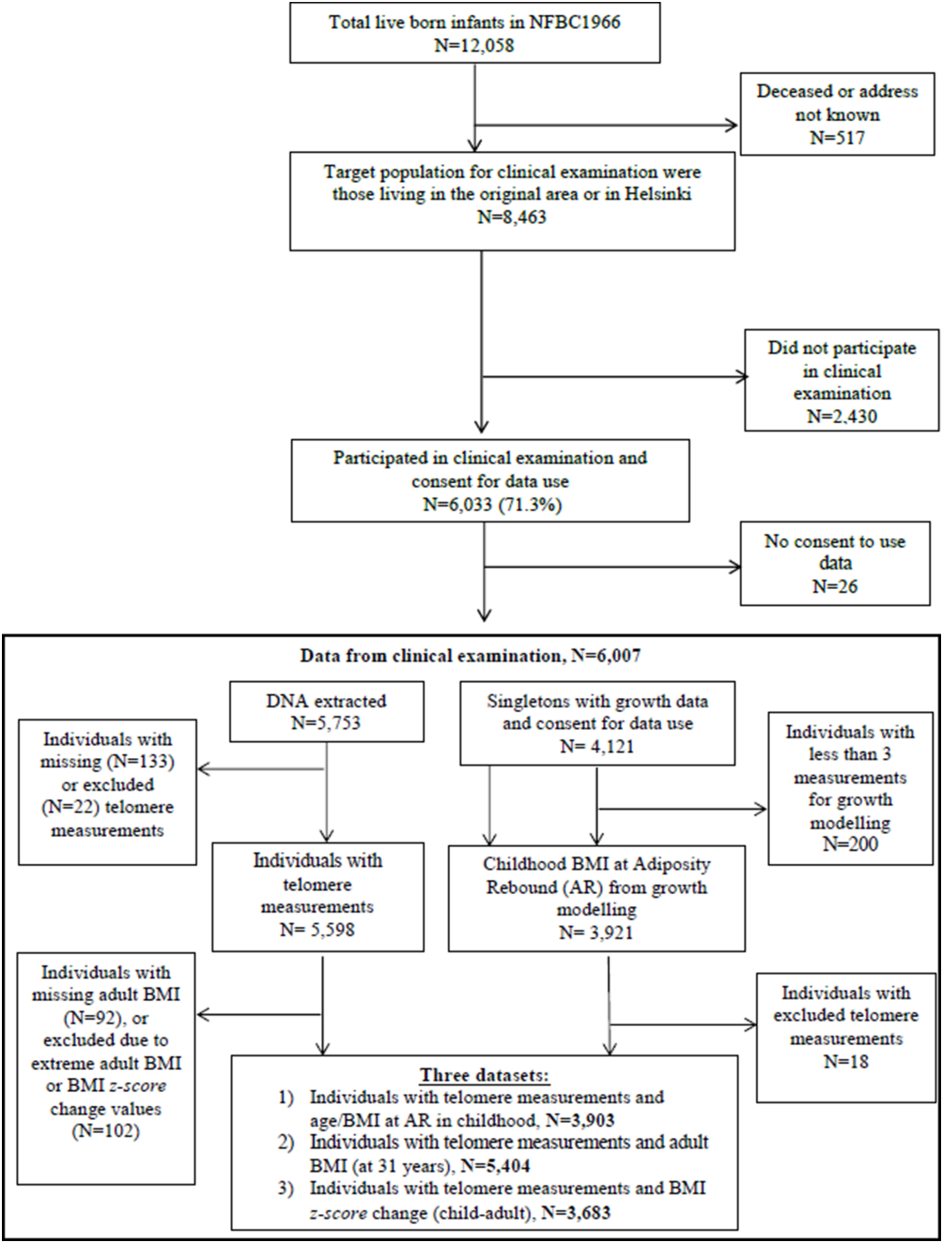
Flow chart for inclusion of study participants.

Pregnant women living in the provinces of Oulu and Lapland with expected delivery dates in 1966 were invited to participate in the NFBC1966, a prospective follow-up study from birth into adulthood [29] (**Figure 1**). A total of 12,058 live-born children (6,169 boys and 5,889 girls) were enrolled in the study; 96.3% of all births in the region. Detailed information on participants at birth, including mother’s parity and parents’ socio-economic status (SES), was collected using questionnaires at hospitals or by trained nurses. Childhood growth measurements were obtained by trained nurses from birth to 16 years of age. At age 31 years, all those alive and with known address were traced (N = 11,541) and sent postal questionnaires, from which information including participant’s children, SES, smoking status and age at menarche was obtained. For this study, smoking status at 31 years was defined as either non/light (<10 cigarettes per day) or heavy (>10 cigarettes per day), since there was no significant difference between the mean telomere length of non-smokers and light smokers (data not shown). A subset of 8,463 living in the original target area or in the Helsinki area were also invited to a clinical examination, in which 6,007 consenting participants took part [30]. Blood samples were taken at that time, from which 5,753 DNA samples were successfully extracted. LTL measurements were obtained for 5,620 of these individuals, of which 22 extreme values were excluded (see below), leaving 5,598 for use in analyses.

### Ethics Statement

Informed written consent for the use of the data and DNA was obtained from all subjects, and approval granted by the Ethics Committee of the Northern Ostrobothnia Hospital District in Oulu (Finland).

### Childhood Anthropometric Measurements

For each individual, predicted age and BMI at AR were calculated using sex-specific polynomial mixed effects models, fitted on BMI measurements collected longitudinally from 18 months to 13 years of age, with the mid-point at 7.25 years. A natural logarithmic transformation of BMI was used to reduce skewness. A detailed description of model selection and fit is given elsewhere [31]. The main model used was as follows: In (BMI (kg/m^2^))  =  β_0_+ β_1_ Age + β_2_ Age^2^+ β_3_ Age^3^+ β_4_ Sex + β_5_ Age * Sex + β_6_ Age^2^ * Sex + u_0_+ u_1_ (Age) + ε.

The β_0_, β_1_, β_2_, β_3_, β_4_, β_5_ and β_6_ are the fixed effects, u_0_ and u_1_ are the individual level random effects and ε is the residual error. Age at AR was defined as the age when BMI measurement reached its minimum, between 2.5 and 8.5 years, with cut-off points chosen from descriptive analysis of growth curves. Each individual’s data were weighted by the number of measurements within the age window to account for uncertainty in the derived parameters. Individuals with fewer than three measurements were excluded from the analysis, as were multiple births.

### Adulthood Anthropometric Measurements

BMI at 31 years was calculated from weight and height measurements using the formula weight/height^2^ (kg/m^2^). Individuals with a BMI<18.5 were defined as underweight, those with a BMI>18.5<25.0 were defined as normal weight, those with a BMI>25.0 were defined as overweight and those with a BMI>30.0 were defined as obese [32].

WHR was calculated from waist and hip measurements taken at age 31 years. BAI at 31 years was calculated as described by Bergman et al [25], as (hip circumference (cm))/((height(m))^1.5^) −18) (**Table 1**).

**Table 1:** Descriptive characteristics of study participants.

	ALL		MALES		FEMALES		
VARIABLES	N (%)	Mean (SD)	N (%)	Mean (SD)	N (%)	Mean (SD)	*P_diff_*
**At birth**							
Maternal parity	5598	2.95 (2.28)	2709	2.93 (2.29)	2889	2.97 (2.26)	0.712
No previous pregnancy	136 (2)		70 (3)		66 (2)		
1 previous pregnancy	1731 (31)		843 (31)		888 (31)		
2 or more previouspregnancies	3731 (67)		1796 (66)		1935 (67)		
Socio-economic statusat birth	5513		2689		2870		0.602
No occupation	1805 (32)		874 (33)		931 (33)		
Farmer	1417 (25)		669 (25)		748 (26)		
White collar	683 (12)		344 (13)		339 (12)		
Blue collar	1654 (30)		802 (30)		852 (30)		
**Childhood (5 y)**							
Age at AdiposityRebound (AR) (years)	3903	5.71 (0.92)	1971	5.81 (0.89)	1932	5.61 (0.95)	<0.001
BMI at AdiposityRebound (AR) (Kg/m^2^)	3903	15.37 (1.07)	1971	15.42 (1.00)	1932	15.31 (1.13)	<0.001
**Adulthood (31 y)**							
Age at menarche (years)	2851				2851	12.92 (1.29)	
Waist-Hip Ratio (WHR)	5398	0.86 (0.08)	2679	0.91 (0.06)	2719	0.81 (0.07)	<0.001
Body mass index (BMI)(kg/m^2^)	5319	24.70 (4.24)	2609	25.23 (3.63)	2710	24.19 (4.69)	<0.001
Underweight	117 (2)		28 (1)		89 (3)		
Normal	3069 (58)		1309 (50)		1760 (65)		
Overweight	1647 (31)		1049 (40)		598 (22)		
Obese	486 (9)		223 (9)		263 (10)		
Body adiposity index (BAI)	5277	25.60 (4.58)	2615	23.01 (2.90)	2662	28.15 (4.50)	<0.001
Adult Smoking	5252		2528		2724		<0.001
Non/Light	4174 (79)		1775 (70)		2399 (88)		
Heavy	1078 (21)		753 (30)		325 (12)		
Socio-economic status	5534		2669		2865		<0.001
Farmer	208 (4)		130 (5)		78 (3)		
Self-employed	1312 (24)		731 (27)		581 (20)		
White collar	3128 (57)		1441 (54)		1687 (59)		
Blue collar	886 (16)		367 (14)		519 (18)		
Children at 31 years	5598	1.26 (1.29)	2709	1.03 (1.21)	2889	1.47(1.33)	<0.001
No children	2184 (39)		1297 (47)		887 (30)		
1 child	1117 (20)		506 (19)		611 (21)		
2 or more children	2297 (41)		906 (34)		1391 (49)		
BMI *z-score* change	3683	−0.01(0.9)	1896	−0.02 (0.9)	1787	−0.01(0.9)	0.812
Leukocyte TelomereLength (LTL)	5598	1.14 (1.40)	2709	1.12 (1.40)	2889	1.17 (1.41)	<0.001

Sample sizes (N) and means and standard deviation (SD) are given for all continuous variables, and sample sizes and category % are given for categorical variables. AR is adiposity rebound; LTL is leukocyte telomere length. WHR is waist-to-hip ratio, BMI is body mass index, calculated as weight (kg)/height (m)^2^. BAI is body adiposity index, calculated as (hip circumference (cm))/((height(m)^1.5^)−18). BMI *z-score* change was calculated separately in males and females as the difference between the *z-scores* of BMI at 31 years, and BMI at AR.

### Longitudinal Change in BMI

Firstly, values for *z-score* BMI at AR in childhood and *z-score* BMI at 31 years were calculated separately in males and females, standardised to zero mean and unit variance. The change in *z-score* BMI was then calculated as the difference between these two measurements: ((BMI *z-score* at 31) − (BMI *z-score* at AR)). The resulting variable, BMI *z-score* change, provides a sex-specific measure of the standardised increase in BMI from childhood to adulthood for each participant.

### Leukocyte Telomere Length (LTL) Measurements

Mean relative LTL was measured in genomic DNA samples prepared from peripheral blood leukocytes taken at age 31, using a multiplex quantitative real-time PCR method [33], with minor modifications as described previously [24]. Briefly, the multiplex qPCR method for measuring mean relative telomere length provides a “T/S ratio” for each DNA sample. This is a relative measure of the amplification of the telomeric DNA sequence (T) compared to that of a single copy gene (S) in each test sample, normalised using a common reference DNA sample. The mean R^2^ values for the calibration curves, based on values obtained for serial dilutions of the reference sample spanning 5–50 ng and run in triplicate, were 0.94 (SD = 0.02) and 0.96 (SD = 0.01) for the T and S amplicons respectively.

The overall mean coefficient of variation (CV) for T/S values of duplicate test samples on the same plate was 5%, and the mean inter-run CV for selected samples was 6.2%.

### Exclusions and Sample Sizes

Individuals with extreme BMI values (BMI>50 kg/m^2^ or a BMI z*-score* change *>*3 standard deviations from the sex-stratified population means, N = 102) were excluded to reduce risk of misclassification due to measurement or data entry errors. Additionally, samples with extreme LTL values were excluded from the analysis (>3 standard deviations from the overall population mean, N = 22). This relatively conservative cut-off was chosen to minimise potential uncertainty about the validity of extreme telomere length values obtained using qPCR [34]. The final number of participants investigated in each part of the study, after exclusions and taking account of all available data, is shown in **Figure 1**. In summary, of the N = 5,753 participants who participated in the clinical examination at age 31 and had DNA samples available, three datasets were selected for analysis. 1) individuals with telomere measurements at 31 years and childhood BMI measurements at AR, N = 3,903; 2) individuals with telomere measurements at 31 years and adult BMI measurements (at 31 years), N = 5,404; and 3) individuals with telomere measurements at 31 years and BMI *z-score* change (age at AR to 31 years), N = 3,683. The samples used for analysis were representative of the NFBC1966 subjects for these parameters and there were no differences observed between the complete dataset and incomplete datasets (*P*>0.05).

### Statistical Analyses

Descriptive characteristics of the study samples were determined overall and for men and women separately (**Table 1**). Means with standard deviation (SD) were calculated for continuous variables, and percentages were calculated for categorical variables, as well as for individuals classed as underweight, normal weight, overweight and obese. Student’s t-test for unpaired data was used to test for differences between males and females, and a Chi-square test was used to test for a difference in percentage distribution in categorical data, with a *P*_diff_ <0.05 considered significant. Kolmogorov-Smirnov tests were performed to determine whether distributions for continuous variables followed normal distributions, and if not then variables were transformed accordingly.

Relative LTL measurements (T/S ratios) were log transformed to achieve normality for use in sex-stratified analyses. LTL was considered as the outcome variable, while age and BMI at AR; WHR, BMI, BAI in adulthood and the BMI *z-score* change were considered as predictor variables. Two models were tested: 1) an unadjusted model, testing for association between log-transformed LTL and adiposity-related measurements from childhood and adulthood; 2) an adjusted model, testing for association between log-transformed LTL and adiposity-related measurements from childhood and adulthood, controlling for potential confounders known to be associated with either the outcome or predictor variables. Those selected were maternal parity and SES at birth (or paternal SES at birth if maternal SES was missing), and age at menarche (females only), SES at 31 years, adulthood smoking and number of children at 31 years. To control for possible batch effects in the telomere measurements, all analyses were also adjusted for qPCR plate. Estimated effects and 95% confidence intervals are reported as % change in LTL per unit change in predictor variable. The Benjamini and Hochberg method was used to address the issue of multiple testing [35], with an adjusted *P* value (*P_corrected_)* of <0.05 considered statistically significant. All statistical analyses were performed using SAS, version 9 (SAS Institute Inc., SAS Campus Drive, Cary, North Carolina 27513, USA).

## Results

### Cohort Characteristics

**Table 1** shows the descriptive statistics for measures of adiposity, LTL and potential confounding variables, for the study population overall and stratified by sex. Consistent with other human population studies [36], we observed a positive association between telomere length and female gender, with men having a mean T/S ratio of 1.12 and women a value of 1.17 (*P*<0.001).

### Association between Childhood Growth and Adiposity Measures and LTL at Age 31

BMI at AR was positively associated with LTL at 31 years in females in unadjusted analyses (*P* = 0.007), and after adjusting for potential confounding factors and correcting for multiple testing (*P_corrected_* = 0.041). There was no association between BMI at AR and LTL at 31 years in males (**Table 2**). There was no evidence for an association between age at AR and LTL at 31 years in either males or females, in unadjusted analyses (Model 1) and after adjusting for potential confounding factors (maternal parity, SES at birth, smoking, SES and having children at 31 years, and additionally, in females only, age at menarche) (Model 2).

**Table 2:** Unadjusted and adjusted association analyses of child and adult adiposity predictors of LTL.

VARIABLES	Unadjusted Model 1				Adjusted Model 2^a^				
	% difference^b^	95% CI		*P*	% difference	95% CI		*P*	*P_corrected_* c
**MEN**									
**Childhood (∼5 years) (N)**	1932				1794				
Age at AR (years)	1.47	−0.24	3.20	0.092	1.22	−0.57	3.05	0.184	0.268
BMI at AR (kg/m^2^)	0.21	−1.28	1.73	0.785	0.40	−1.20	2.02	0.625	0.682
**Adulthood (31 years) (N)**	2662				2423				
Waist-hip ratio (WHR)	−0.44	−0.79	−0.09	0.014	−0.42	−0.79	−0.04	0.029	**0.049**
BMI (kg/m^2^)	−29.51	−43.31	−12.35	0.002	−26.86	−41.97	−7.80	0.008	**0.025**
Body adiposity index (BAI)	−0.25	−0.76	0.13	0.164	−0.29	−0.76	0.18	0.223	0.268
**BMI ** ***z-score*** ** change, childhood** **to adulthood (N)**	1896				1730				
	−0.98	−2.58	0.65	0.238	−1.08	−2.78	0.64	0.218	0.268
**WOMEN**									
**Childhood (∼5 years) (N)**	1971				1774				
age at AR (years)	−0.12	−1.69	1.48	0.882	−0.33	−2.07	1.44	0.712	0.712
BMI at AR (kg/m^2^)	1.84	0.49	3.21	0.007	1.71	0.26	3.18	0.020	**0.041**
**Adulthood (31 years) (N)**	2742				2517				
Waist-hip ratio (WHR)	−0.51	−0.78	−0.23	<0.001	−0.50	−0.79	−0.21	<0.001	**0.008**
BMI (kg/m^2^)	−23.04	−35.82	−7.71	0.005	−22.03	−35.76	−5.37	0.012	**0.029**
Body adiposity index (BAI)	−0.48	−0.71	−0.14	0.004	−0.44	−0.74	−0.13	0.005	**0.021**
**BMI ** ***z-score*** ** change, childhood** **to adulthood (N)**	1787				1640				
	−3.08	−4.74	−1.40	<0.001	−2.91	−4.65	−1.15	0.001	**0.008**

AR is adiposity rebound; LTL is leukocyte telomere length. WHR is waist-to-hip ratio, BMI is body mass index, calculated as weight (kg)/height (m)^2^. BAI is body adiposity index, calculated as (hip circumference (cm))/((height(m)^1.5^)−18). BMI *z-score* change was calculated separately in males and females as the difference between the *z-scores* of BMI at AR, and BMI at 31 years. The number of individuals in each analysis is given (N); those in adjusted analyses are slightly lower than corresponding unadjusted analyses due to missing data for one or more covariates.

a Linear regression model adjusted for maternal parity, SES at birth, Smoking at 31 years, SES at 31 years, children at 31 years and qPCR plate. In addition to these potential confounders, the model was also adjusted for age at menarche in women.

b % change in LTL per unit change in predictor variable.

c Statistically significant *P_corrected_* values are shown in **bold**. FDR *P_corrected_* values were calculated after adjustment using the Benjamini-Hochberg procedure [34], which provides a correction for multiple testing.

### Association between Adulthood Adiposity-related Phenotypes and LTL at Age 31

In the unadjusted Model 1, WHR was inversely associated with LTL at 31 years in both males (*P* = 0.014) and females (*P*<0.001). After adjustment for potential confounders (maternal parity and SES at birth, age at menarche (female participants), SES, smoking and number of children at 31 years) and correction for multiple testing (Model 2), the association remained significant in females (*P_corrected_*  = 0.008) and males (*P_corrected_*  = 0.049). An inverse association between BMI and LTL at 31 years was also observed in both males (*P* = 0.002) and females (*P* = 0.005), which remained significant in males (*P_corrected_* = 0.025) and females (*P_corrected_* = 0.029) after adjustment for potential confounders and correction for multiple testing (Model 2). Body fat percentage, estimated by BAI at age 31 was also inversely associated with LTL in females (*P* = 0.004) and remained significant after adjustments (*P_corrected_* = 0.021). However, BAI was not associated with LTL in males (**Table 2**).

### Association between BMI *z-score* Change from Childhood-adulthood and LTL at 31

BMI *z-score* change from AR in childhood (at around 5 years) to 31 years was inversely associated with LTL at 31 years in females (*P*<0.001) but not males (*P* = 0.238) (Model 1). A one unit increase in BMI *z-score* change was associated with a 3.08% decrease in LTL in females (Model 1), which reduced slightly to 2.91% after adjustment for potential confounding factors (*P_corrected_* = 0.008).

## Discussion

We have shown in a large population-based cohort that a greater increase in standardised BMI from early childhood to adulthood is associated with shorter mean relative LTL in women aged 31. We have also shown that BMI and WHR at age 31 are inversely associated with adult LTL in both men and women in this study, and that higher BAI is inversely associated with LTL in women only. All these associations remain significant after adjusting for multiple potential confounders (maternal parity and SES at birth, and age at menarche (females only), SES, smoking and number of children at 31 years) and correcting for multiple testing. Our results thus confirm and extend those reported in a recent meta-analysis of BMI and LTL [23].

Our finding that WHR and BMI are both associated with LTL in adult women is consistent with previous reports of an inverse relationship between measures of adiposity and concurrent telomere length [8], [16], [19], [20], [37]. In contrast to Nordfjall et al [37], we found that BMI is associated with shorter telomeres in adult men as well as in women. In addition, we identified an inverse association between WHR and LTL in adult men, which is consistent with a previous report that WHR is a significant predictor of faster telomere shortening rate in a predominantly male cohort (>80%) [17]. To our knowledge, our study is the first to show that LTL is also inversely associated with BAI in women. We did not see any evidence for an association between BAI and LTL in men, perhaps because BAI may be more strongly correlated with percentage of body fat in women than in men [38].

The gender difference for our main finding - that a greater increase in standardised BMI from childhood to 31 years is associated with shorter adult LTL - may reflect the fact that a greater proportion of change in BMI in women compared to men over this period is characterised by increased body fat [39]. This association remains significant even after adjusting for number of children born to participants by age 31 years, suggesting that this finding is unrelated to pregnancy.

We did not find any evidence for an association between age at adiposity rebound (AR) and LTL at age 31 in either sex, despite previous studies in this cohort and others showing that earlier age at AR predicts higher BMI and poorer metabolic health in adulthood [28], [40]. Intriguingly, we identified a *positive* association between BMI at AR and adult telomere length in women, which remained significant after correcting for maternal parity and SES at birth, and age at menarche, SES, smoking status and number of children at 31 years. Furthermore, there is no evidence of association between BMI at AR and cardiometabolic profiles at age 31 in this cohort [40]. We speculate that these findings may reflect a beneficial effect of having a childhood BMI within the normal range (as did the majority of participants in this population cohort) on adult telomere length and metabolic health. Further longitudinal studies of growth throughout childhood, weight gain in adulthood and telomere measurements taken at multiple time points are required to elucidate the mechanisms underlying these observations.

The major strengths of our study include the uniform age and ethnicity of the participants, the even sex ratio and the availability of LTL data for a large number of samples. Although concerns have previously been expressed over the reliability of the qPCR method [34], the telomere measurements used in this study show the expected sex difference, and have previously been used in the identification of robust genetic associations with LTL [4]. Additionally, the use of growth modelling phenotypes allowed the investigation of the relationship between important measurements of childhood adiposity and LTL in adulthood.

Due to the availability of DNA samples for only one time-point, it was not possible to study the association between longitudinal telomere length change and BMI trajectories over the same period. Thus, we were unable to draw any conclusions regarding causality for the relationship between BMI increase and telomere length. We were also unable to study the potential effects of interim changes in BMI between childhood and adulthood. Finally, it would have been interesting to further investigate the effects of underweight and obesity in childhood and adulthood on adult LTL, but we did not have sufficient numbers of such individuals available in this population cohort.

Despite these limitations, our study adds to the growing evidence supporting the hypothesis that factors associated with obesity, such as increased systemic levels of oxidative stress and/or inflammation, may have similar effects on lifespan and cellular function to the ageing process [41]. Our findings warrant further investigation of the potential for using telomere length as a biomarker to assess the effectiveness of interventions to combat the negative impact of obesity on healthy ageing. These include increased moderate physical activity, which has been associated with longer telomeres (reviewed in [42]), as have dietary and holistic weight loss interventions [43], [44].

## Conclusion

We show that LTL is inversely associated with multiple measures of adiposity in both men and women, and that BMI increase in women from childhood to adulthood is associated with shorter telomeres at age 31. Further longitudinal studies are now required to investigate the long-term effects of weight gain after childhood on LTL, healthy ageing and disease risk, and to investigate the gender differences observed.
